# Long-Term Survival in Metachronous Primary Malignancies: Stage III Nasopharyngeal Cancer and Stage IV Non-Small-Cell Lung Cancer

**DOI:** 10.3390/jcm14103299

**Published:** 2025-05-09

**Authors:** Gabriela Rahnea-Nita, Alexandru Nechifor, Mihai-Teodor Georgescu, Dorel Firescu, Adrian-Cornel Maier, Radu-Valeriu Toma, Valentin Titus Grigorean, Liliana-Florina Andronache, Roxana-Andreea Rahnea-Nita, Ionut Simion Coman, Laura-Florentina Rebegea

**Affiliations:** 1 Specific Disciplines Department, Faculty of Nursery and Midwifery, “Carol Davila” University of Medicine and Pharmacy, 020021 Bucharest, Romania; gabriela.rahnea-nita@umfcd.ro; 2 M Hospital—M Care, 077190 Voluntari, Romania; 3 Clinical Department, Faculty of Medicine and Pharmacy, “Dunarea de Jos” University of Galati, 800008 Galati, Romania; alexandru.nechifor@ugal.ro (A.N.); dorel.firescu@ugal.ro (D.F.); adrian.maier@ugal.ro (A.-C.M.); laura.rebegea@ugal.ro (L.-F.R.); 4 Clinical Department, Faculty of Medicine, “Carol Davila” University of Medicine and Pharmacy, 020021 Bucharest, Romania; mihai.georgescu@umfcd.ro (M.-T.G.); radu.toma@umfcd.ro (R.-V.T.); valentin.grigorean@umfcd.ro (V.T.G.); 5 Department of Radiotherapy, Oncological Institute “Prof. Dr. Alexandru Trestioreanu”, 022328 Bucharest, Romania; 6 Department of General Surgery, “Bagdasar-Arseni” Clinical Emergency Hospital, 041915 Bucharest, Romania; 7 Preclinical Department, Faculty of Medicine, “Carol Davila” University of Medicine and Pharmacy, 020021 Bucharest, Romania; liliana.andronache@umfcd.ro; 8 M Hospital—M Oncology, 077190 Voluntari, Romania; 9 Department of Radiotherapy, “Sf. Apostol Andrei” Hospital, 800578 Galati, Romania

**Keywords:** metachronous primary cancers, nasopharynx, lung cancer, long-term survival, favorable response

## Abstract

**Introduction**: The occurrence of a second primary lung cancer after head and neck cancer is a challenge for multidisciplinary teams, since the development of a second lung cancer negatively affects the survival rate of patients with head and neck cancer. **Case Presentation**: This article presents the case of a patient with a double location of cancer: inoperable stage III nasopharyngeal carcinoma, biopsied in December 2017 (non-keratinizing nasopharyngeal carcinoma), treated by means of radiotherapy and chemotherapy (2018–2021), and stage IV lung cancer (squamous carcinoma) with lung metastases, diagnosed in December 2021, treated using polychemotherapy, subsequent maintenance monochemotherapy, radiotherapy of the thorax, and subsequent maintenance monochemotherapy with a favorable result. The patient was still under treatment as of February 2025, the date of the preparation of the current article. **Discussion and Literature Review**: Regarding the location of the second metachronous cancer, studies show that the most frequent locations are the lungs and the esophagus, with the main causes being alcohol consumption and smoking. Therefore, these patients should be monitored by screening the respiratory and digestive tracts, especially in men, in order to identify a second cancer, either synchronous or metachronous, in an early stage. **Conclusions**: Educating the patient with head and neck cancer regarding quitting smoking and cutting out alcohol, as well as conducting a follow-up survey, may reduce the incidence of multiple primaries. Moreover, the multidisciplinary management of second primary lung malignancies in patients with head and neck cancer may lead to long-term disease monitoring.

## 1. Introduction

Metachronous primary malignancies (MPMs) refer to the occurrence of multiple primary cancers in the same patient at different time points, with at least a six-month interval between diagnoses. Advances in cancer diagnostics and treatment have increased MPM cases due to prolonged survival and improved detection methods [1,2]. However, managing MPMs presents significant clinical challenges, as treatment strategies must consider tumor heterogeneity, patient tolerance, and potential interactions between therapeutic modalities [3].

Nasopharyngeal carcinoma (NPC) is a relatively rare malignancy, but its incidence is notably higher in specific geographic regions, particularly Southeast Asia and parts of North Africa [4]. NPC is often associated with Epstein–Barr virus (EBV) infection, and its treatment primarily involves radiation therapy and chemotherapy [5]. On the other hand, non-small-cell lung cancer (NSCLC) accounts for approximately 85% of all lung cancer cases and remains one of the leading causes of cancer-related mortality worldwide [6]. The prognosis of stage IV NSCLC is particularly poor, with limited long-term survival despite advances in immunotherapy and targeted treatments [7].

The occurrence of NPC and NSCLC as metachronous malignancies in the same patient is exceedingly rare. While previous reports have documented synchronous or metachronous lung cancers following head and neck malignancies, the mechanisms underlying these associations remain unclear [8,9]. Some hypotheses suggest genetic susceptibility, shared environmental carcinogens (e.g., smoking, radiation exposure), and treatment-induced secondary malignancies as possible contributing factors [10,11].

In this case report, we present a patient with long-term survival following the sequential diagnosis of stage III NPC and stage IV NSCLC. This case is significant because it highlights the complex interplay between cancer biology, treatment decisions, and patient resilience. By detailing the clinical course, therapeutic strategies, and long-term outcomes, we aim to contribute valuable insights to the management of MPMs and provide a reference for future cases [12].

## 2. Case Presentation

This article presents the case of a 55-year-old male patient at the time of diagnosis in 2017, with a double location of cancer: inoperable rhino-pharyngeal neoplasm with extension to the base of the cranium, with bilateral latero-cervical adenopathies, biopsied in December 2017 (non-keratinized nasopharyngeal carcinoma), and right lung cancer (squamous carcinoma) with lung metastases, diagnosed through biopsy and CT scan in November 2021.

The patient has been a smoker since the age of 17, smoking two packs of cigarettes a day until 2017 when he got sick and quit both smoking and alcohol consumption. The patient was a bus driver and stated that in 2010, which is 7 years before the diagnosis, he underwent severe polytrauma to the nose, being hit by the door of the bus hold for luggage.

In terms of comorbidities, the patient suffers from chronic B hepatitis virus (HBV) with the Delta agent, diagnosed in 2010.

The first symptom of nasopharyngeal cancer was otalgia in 2017.

Throughout his oncologic evolution, the patient has been diagnosed with a severe form of SARS-CoV-2 infection in December 2020, chronic venous disease of the lower limbs in 2019, deep venous thrombosis in 2019, and chronic non-specific trochanteric osteomyelitis with *Staphylococcus aureus*, treated with antibiotics, in May 2022.

The patient is under chronic treatment with oral anticoagulants.

**Figure 1:**
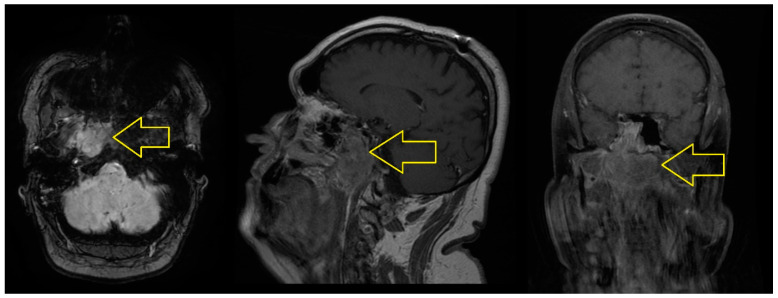
MRI of the cranio-cerebral area with contrast medium in November 2017. The infiltrating tumor mass affects the cavity on the right side, with extension at the base of the cranium (yellow arrow).

In November 2017, the cranio-cerebral MRI scan with contrast medium revealed the following: “A tumor mass located in the right sided cavity, with extension to the left medial line, with axial diameters of 35/43 mm and cranio-caudal diameters of 41 mm, with irregular outlines; this determines the erasure of the mucosal outline at this level, with the involvement of the tensor muscles of the palatine wall and upper constrictor of the pharynx, it impacts the cellular-adipose structures corresponding to the right parapharyngeal space, respectively, it presents a posterior extension of the retropharyngeal space on the right side, thus affecting the longus capitus muscle, as well as upper extension at the base of the cranium, where it has an osteolytic character, by impacting the basisphenoid, the large wing of the sphenoid bone, and the apex of the temporal rock on the right side; moreover, the tumor mass partially impacts the carotid canal, with a sleeve of the right internal carotid artery in the intrapetrous segment, and at the level of the upper pole it presents an extension to the right sphenoidal hemisinus, as well as a slight extension in the right middle cerebral fossa, coming in contact with the antero-infero-medial slope of the right temporal lobe and with the lower slope of the area of the right venous cavernous sinus. Isolated adenopathies located in the upper and middle floor of the jugular-carotid level, superficial parotid, submandibular and posterior cervical, on the right side, respectively, at the level of the middle floor of the jugular-carotid level and posterior cervical on the left side, the largest of 17/11 mm in the middle floor of the jugular-carotid level on the right side” (Figure 1).

A biopsy was performed in December 2017, and the histopathological and immunohistochemical examinations revealed the diagnosis of non-keratinized nasopharyngeal carcinoma (CK5 positive in the tumor proliferation; CK7 negative; p63 slightly positive in the tumor cells, index Ki-67 approx. 20%).

Determination of nasopharyngeal cancer stage: T3 N2 M0: stage III.

For the nasopharyngeal cancer with extension at the base of the cranium, which had bilateral latero-cervical adenopathies, the patient underwent external 3D conformational radiotherapy (ERT) in 35 fractions, with a dose/fraction = 2 Gy and a total dose = 70 Gy, 5 days/week, between January and March 2018. In addition, they were treated with Cetuximab plus Carboplatin (initial Cetuximab dose of 400 mg/m^2^ IV over 2 h, followed by weekly Cetuximab doses of 250 mg/m^2^ IV over 1 h, and then Carboplatin AUC 5 mg·min/mL IV, in a process that was repeated every 3 weeks, amounting to six cycles ending in April 2018), and then received maintenance therapy with Cetuximab (Cetuximab 250 mg/m^2^ IV over 1 h weekly) until October 2021.

**Figure 2:**
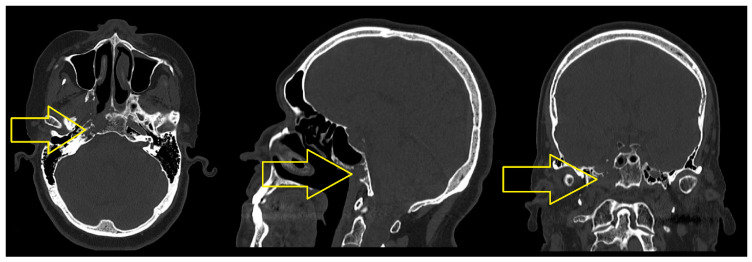
The computed tomography of the cervical area with contrast medium—October 2019. The nasopharyngeal tumor with osteolysis at the base of the cranium (yellow arrow), with extension of 30/20/15 mm.

The CT scan of the cervical region performed in October 2019 revealed the following: “the osteolytic lesion at the base of the brain with an extension of 30/20/15 mm, affecting the sphenoid body, the lateral walls and inferiorly the right sphenoid sinus and the tip of the temporal rock, in a minimal regression from the previous CT scan in April 2019. The latero-cervical and submandibular lymph nodes are reduced in dimension, with no tumor indication” (Figure 2).

The CT scans performed in May and November 2020 revealed that the nasopharyngeal tumor was dimensionally stable, with osteolysis of the base of the cranium in minimal regression, and there were no latero-cervical adenopathies.

**Figure 3:**
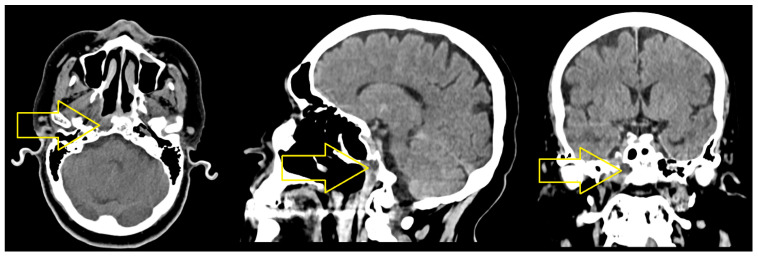
The CT scan of the cervical area with contrast medium—October 2021. Moderate diffuse thickening of the mucosa of the cavity of 5 mm (yellow arrow).

**Figure 4:**
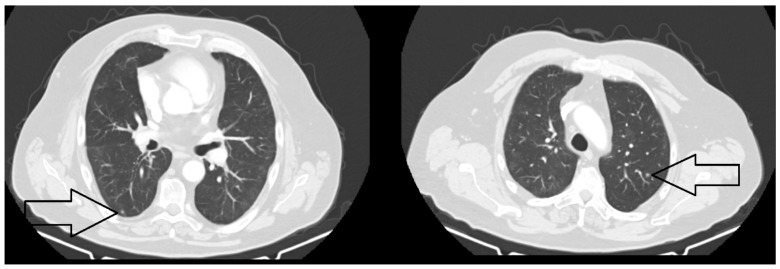
The CT scan of the thorax with contrast medium—October 2021. A few newly occurring micronodules and small pulmonary nodules (black arrows) appeared in the right Fowler segment, respectively, of the upper lingula (9/7 mm).

After 11 months, the computed tomography assessment of the cervical area, head, thorax, and upper abdomen performed in October 2021 revealed the following: “Moderate diffuse thickening of the mucosa of the cavity of 5 mm, right submandibular adenopathy (9/7 mm), no cervical adenopathies. A few newly occurred micronodules and small pulmonary nodules at the level of right Fowler segment, respectively, of the upper lingula (9/7 mm)” (Figure 3 and Figure 4).

Following the CT scan of the thorax performed in October 2021, a bronchoscopy was recommended, which was performed in November 2021, revealing a budding formation in the well-vascularized right middle lobe, which subtotally obstructed the middle lobe and prevented the bronchoscope from being inserted. Serial biopsies were performed, and the histopathological diagnosis was as follows: squamous G2 cell carcinoma, with bronchial location.

The results of the immunohistochemical tests in December 2021 revealed the presence of pulmonary squamous cell carcinoma, which was EGFR-negative, ALK-negative, and PDL1-negative.

The therapeutic indication board, reunited in December 2021, recommended beginning chemotherapy with Carboplatin plus Docetaxel (Carboplatin AUC 5 and Docetaxel 75 mg/m^2^ administered once every 3 weeks) across eight series to treat the stage IV lung cancer with lung metastases.

The MRI scans performed in January and February 2022 and the PET CT scan revealed an osteolytic tumor mass at the left peritrochanteric level, in progression, for which an incisional biopsy was performed in March 2022; the histopathological tests established the occurrence of non-specific chronic osteomyelitis with *Staphylococcus aureus*, for which the patient underwent a course of antibiotic treatment.

**Figure 5:**
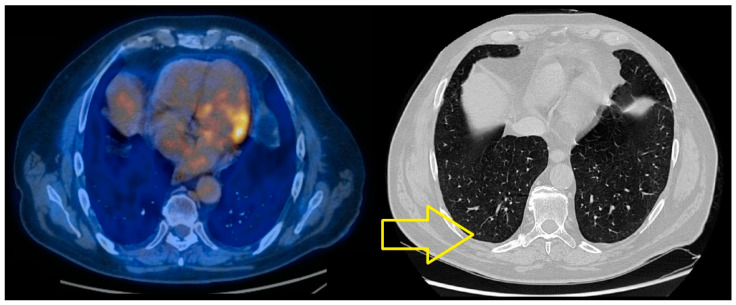
PET-CT scan—June 2022. Dimensional regression of the bilateral micronodular and nodular lesions, with a current maximum of 3 mm (yellow arrow).

The PET-CT scan performed in June 2022 revealed a dimensional regression of the bilateral micronodular and nodular lesions, with a current maximum of 3 mm, compared to 7/6 mm previously, as established through secondary determinations (Figure 5).

The CT scan of the brain, cervical area, thorax, abdomen, and pelvis performed in September 2022 revealed a tumor mass in the cavity with osteolysis of the right hemiclivus and the lateral wall of the right compartment of the sphenoid sinus measuring 20/17 mm, which was dimensionally stable from the previous examination. The previously described densified micronodules were no longer identifiable in the right pulmonary lobe.

Since 2022, the patient has undergone maintenance monochemotherapy with Docetaxel (Docetaxel 75 mg/m^2^ administered once every 3 weeks).

The CT scan of the cervical area, thorax, abdomen, and pelvis performed in March 2023 revealed a tumor mass in the cavum measuring 20/17 mm, with no pulmonary nodules and no hilar or mediastinal adenopathies.

A PET-CT scan performed in May 2023 revealed metabolically active mediastinal adenopathies, for which a mediastinoscopy was performed in June 2023, revealing anthracosis.

The bronchoscopy performed in July 2023 revealed neoplastic cells.

In August 2023, the patient underwent external photon radiotherapy using the VMAT technique (total dose = 30 Gy, 10 fractions/2 weeks, with dose/fraction = 3 Gy on PTV (planning target volume)-positive adenopathies from the PET-CT scan), and the treatment was well tolerated.

The CT scan performed in September 2023 revealed mediastinal adenopathies. The biopsy performed in October 2023 revealed the following: squamous cell carcinoma, for which the patient underwent switch maintenance monochemotherapy with Gemcitabine (Gemcitabine 1000 mg/m^2^ administered on days 1 and 15). The decision was made to administer on days 1 and 15 and not 1, 8, and 15 because the patient had been receiving ongoing oncology treatments for 8 years, and there was an increased risk of cumulative chemotherapy and radiation toxicity. The hematopoietic growth factor pegfilgrastim was also prescribed.

In March 2024, a CT scan was performed and the patient continued the treatment with another switch in maintenance therapy, namely, monochemotherapy with Vinorelbinum in tablet form (60 mg/m^2^, weekly for the first 3 weeks, and then 80 mg/m^2^, weekly for the next 6 weeks).

The results of the bronchoscopy performed in May 2024 were negative for malignancies. In September 2024, the patient repeated the CT scan, and the bronchoscopy in November 2024 revealed neoplastic cells.

**Figure 6:**
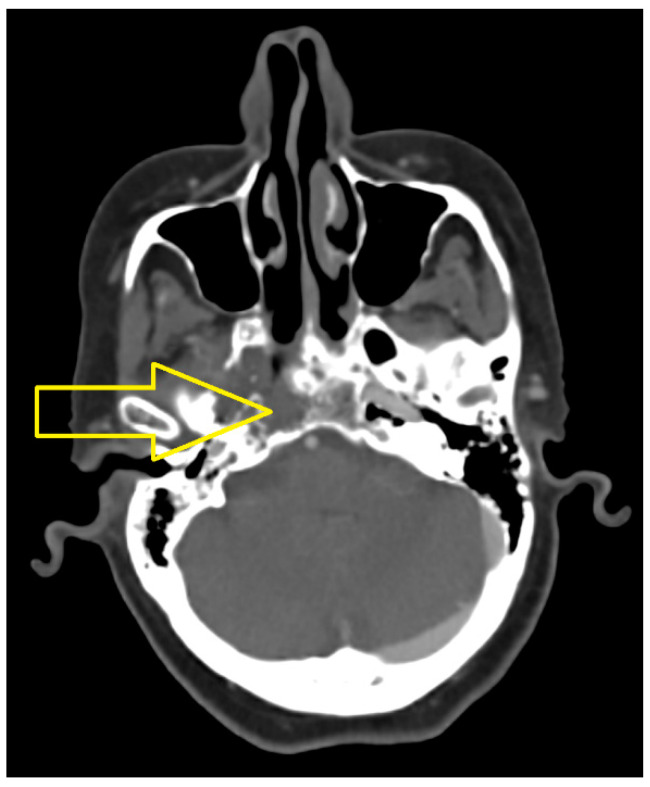
CT scan of the cervical region—December 2024. The focal lysis was maintained in the cavum (yellow arrow). No submandibular or latero-cervical lymphadenopathies.

**Figure 7:**
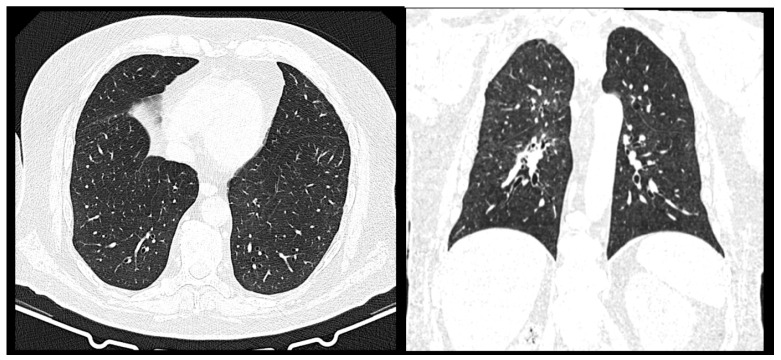
CT scan of the thorax—December 2024. No newly occurring or evolutionary pleuro-pulmonary nodular lesions.

Upon the thoracic surgery examination performed in December 2024, it was recommended that the CT scan was repeated, which revealed a stationary aspect with no signs of local relapse or unfavorable evolution (the focal lysis was maintained in the cavum, no individualized tumor tissue was revealed upon the CT scan, and no newly occurring or evolutionary nodular lesions were present within the lungs) (Figure 6 and Figure 7).

Therefore, upon the thoracic surgical examination, it was decided that the patient did not require surgery and the best option in terms of risks/benefits was to continue the oncological treatment.

So, due to the presence of neoplastic cells, revealed in December 2024, it was decided that the patient must reinitiate chemotherapy, with the chosen agent being Gemcitabine (Gemcitabine 1000 mg/m^2^ administered on days 1 and 15).

Since December 2024, the patient has continued the maintenance monochemotherapy with Gemcitabine (Gemcitabine 1000 mg/m^2^ administered on days 1 and 15). This treatment was still being administered in February 2025 (the time when the current article was prepared), and the patient’s performance status was ECOG = 1.

It is important to note that despite the fact that so many chemotherapy agents were administered, the patient never experienced changes in their laboratory analyses, and also, except for the rash caused by Cetuximab, no other symptoms were revealed. He tolerated all the treatments very well.

Given the complexity of this case, a summary of the clinical evolution is provided in Table 1, detailing the timeline of diagnoses, key clinical findings, treatments administered, and patient outcomes. This helps to illustrate the interplay between multiple malignancies and their management over time.

## 3. Discussion and Literature Review

We reviewed the most relevant articles published and we selected ten studies with a clinical impact, published in the past six years.

The main updates include insights into metachronous cancer, risk factors, survival rates, and follow-up surveys.

These selected articles are synthesized and listed in Table 2.

In an article published in 2022, Pan, S-Y. et al. [1] identified key risk factors associated with multiple primary tumors, including viral infections, chemotherapy, radiation, and genetic predisposition. Identifying these risk factors in patients with a primary malignancy may facilitate early detection of a second primary cancer, leading to better clinical outcomes [13].

Accidental detection of a second primary malignancy most frequently involves hepatocellular carcinoma, renal carcinoma, lung carcinoma, or bladder carcinoma [14]. Elicin, O. et al. (2019) [14] retrospectively analyzed 296 patients with head and neck cancer who underwent radiotherapy combined with chemotherapy using platinum salts or monoclonal antibodies (Cetuximab). The study found that patients who switched from platinum compounds to Cetuximab due to toxicity had a higher risk of developing a second primary cancer than those treated exclusively with either platinum-based chemotherapy or Cetuximab [15].

Regarding surgical interventions, Yankov G et al. (2023) [10] described a case of a patient with primary squamous cell laryngeal carcinoma (T4N2M0, G1) treated with laryngectomy and chemoradiotherapy, who later developed metachronous squamous cell carcinoma of the lung (pT3pN2M0, G2) and underwent pneumonectomy [16]. The literature suggests that the lungs and esophagus are the most common sites for second metachronous malignancies, with alcohol consumption and smoking being primary risk factors [17,18]. Consequently, screening of the respiratory (e.g., low-dose CT scan or PET-CT) and digestive tracts (e.g., endoscopy), particularly in men, is recommended for early detection of synchronous or metachronous tumors [19].

A study by Mot, I-C. et al. (2019) [18] found that the most common second primary malignancies were skin cancers, specifically basal cell carcinoma and squamous cell carcinoma [20]. The prognosis of patients with metachronous cancer is significantly influenced by whether additional malignancies develop. When the second primary cancer is located in the lungs or esophagus, survival rates tend to be lower [21,22]. While some studies suggest that patients with metachronous cancers have better survival outcomes than those experiencing recurrence of head and neck cancer, other studies indicate that a second malignancy is the primary cause of long-term mortality [20,23,24].

A retrospective study conducted in France by Clément-Duchêne, C. et al. (2002–2016) [23] included 25 patients diagnosed with head and neck cancer and early or locally advanced lung cancer. The majority of patients were male smokers and alcohol consumers, with a median survival of 41.9 months [25]. In another study, Ha, J. et al. (2019) [24] reported a lower incidence of secondary primary malignancies compared to previous research but confirmed that progression to synchronous or metachronous cancer was associated with significantly worse overall survival [26]. Similarly, a study by Eui Bae Kim et al. (2008) [25] emphasized that patients with head and neck cancer have an increased risk of developing a second primary lung cancer, recommending routine chest CT or PET-CT scans for early detection [27].

**Table 2:** Comparative analytical data.

No.	Author, Year	Subject	Reference No.
1	Pan, S.-Y. et al., 2022	Review of associated risk factors	[1]
2	Corvino, A. et al., 2020	Prevalence and patterns of occurrence in multidetector computed tomography	[13]
3	Elicin, O. et al., 2019	Second primary cancers after radiotherapy combined with platinum and/or Cetuximab in head and neck cancer	[14]
4	Yankov, G. et al., 2023	Metachronous second primary lung cancer after laryngectomy for laryngeal carcinoma	[10]
5	Bugter, O. et al., 2019	Survival of patients with head and neck cancer with metachronous multiple primary tumors	[15]
6	Moț, I-C. et al., 2019	Histopathological and immunohistochemical study	[18]
7	Iwatsubo, T. et al., 2019	Impact of age at diagnosis of head and neck cancer on incidence of metachronous cancer	[19]
8	Clément-Duchêne, C. et al., 2021	The epidemiology of head and neck cancers and the association with lung cancer	[23]
9	Ha, J. et al., 2019	Synchronous and metachronous head and neck squamous cell carcinoma in a single center	[24]
10	Tapati Mandal et al., 2021	Early detection of the presence of a second primary malignancy	[26]

Tapati Mandal et al. (2021) [26] presented a case in which a patient with pyriform sinus carcinoma was diagnosed with metachronous lung cancer using 18F-FDG PET-CT. Their study reinforced the role of PET-CT as an effective method for early detection of secondary primary malignancies, even in asymptomatic patients [28].

For non-small-cell lung cancer (NSCLC) patients who are EGFR-negative, ALK-negative, and PDL1-negative, standard treatment consists of multiple cycles of chemotherapy, often incorporating platinum-based agents. Maintenance therapy strategies, such as continuation maintenance (prolonging treatment with agents from the initial regimen) or switch maintenance (introducing a new non-cross-resistant drug), have demonstrated benefits in prolonging progression-free and overall survival [29,30,31].

The case conclusions are as follows:

Lung cancer was identified by serial biopsies that were performed; the histopathological results indicated the presence of squamous G2 cell carcinoma, with bronchial location. The results of the immunohistochemical tests in December 2021 revealed the presence of pulmonary squamous cell carcinoma, which was EGFR-negative, ALK-negative, and PDL1-negative. Also, the imaging techniques, CT scans, and PET-CT scans corroborated, in real time, the good response to lung cancer treatment.

Disease control was first revealed after the first-line treatment with Docetaxel and Carboplatin for lung cancer. The PET-CT scan performed in June 2022 revealed a dimensional regression of the bilateral micronodular and nodular lesions, with a current maximum of 3 mm, compared to 7/6 mm for the previous metastases. Thereafter, while undergoing maintenance therapy with Docetaxel, the CT scan of the brain, cervical area, thorax, abdomen, and pelvis, performed in September 2022, revealed a tumor mass in the cavity with osteolysis of the right hemiclivus and the lateral wall of the right compartment of the sphenoid sinus measuring 20/17 mm, which was dimensionally stable, as assessed through comparison with the previous examination. The previously described densified micronodules are no longer identified in the right pulmonary lobe. Thereafter, the CT scan of the cervical area, thorax, abdomen, and pelvis performed in March 2023 revealed a tumor mass in the cavum measuring 20/17 mm, with no pulmonary nodules and no hilar or mediastinal adenopathies. Basically, a complete response to the thoracic disease was sustained, as shown by the PET-CT scan performed in May 2023, which revealed metabolically active mediastinal adenopathies, for which a mediastinoscopy was performed in June 2023, revealing anthracosis. This complete response was then overturned by a bronchoscopy that revealed neoplastic cells, for which the patient underwent radiotherapy, and then changed to switch maintenance with Vinorelbinum and Gemcitabine, as a result of another bronchoscopy that revealed neoplastic cells.

The only remaining signs of the nasopharyngeal cancer within the last few years are the tumor mass of the cavity with osteolyisis of the right hemiclivus and the lateral wall of the right compartment of the sphenoid sinus measuring 20/17 mm; therefore, we are discussing administering an appropriate biopsy to figure out a differential diagnosis.

The distinctive aspects of our case include the following:

- The diagnosis of both cancers at an advanced stage (stage III nasopharyngeal cancer in December 2017 and stage IV lung cancer in December 2021).
- A time interval of four years between the two diagnoses.
- A long-term favorable response of nasopharyngeal cancer to treatment, with continued responsiveness as of December 2024. The patient received radiotherapy followed by polychemotherapy (Cetuximab plus Carboplatin every three weeks for six cycles in April 2018), with Cetuximab maintenance therapy continuing until October 2021.
- The decision to perform bronchoscopy to distinguish between primary lung cancer with metastases versus lung metastases originating from nasopharyngeal carcinoma, despite no clear evidence of a primary lung tumor on CT scan. This decision was guided by the suspicious appearance of lung micronodules, the history of favorable response of nasopharyngeal carcinoma, and the known increased risk of secondary lung malignancies in patients with head and neck cancer.
- The complete resolution of lung micronodules after eight cycles of polychemotherapy with Carboplatin and Docetaxel, as confirmed by thoracic CT in September 2022.
- The continued favorable response of metastatic lung cancer to treatment as of December 2024, with no signs of local relapse. The use of first-line chemotherapy (Carboplatin plus Docetaxel), continuation maintenance chemotherapy (Docetaxel), and switch maintenance chemotherapy (Vinorelbine and Gemcitabine) has played a crucial role in achieving prolonged progression-free and overall survival.
- The differential diagnosis of an osteolytic tumor mass in the left peritrochanteric region was clarified through MRI and PET-CT scans performed in January and February 2022. An incisional biopsy in March 2022 confirmed chronic osteomyelitis with Staphylococcus aureus, for which targeted antibiotic therapy was administered.
- A biopsy of metabolically active mediastinal adenopathy identified on PET-CT in May 2023 revealed anthracosis.
- As of February 2025, the patient has an ECOG performance status of 1, enjoys an excellent quality of life, and has survived for over eight years since the diagnosis of nasopharyngeal cancer and three years since the diagnosis of metastatic lung cancer.

This case underscores the importance of long-term surveillance, individualized treatment strategies, and early intervention in detecting and managing secondary primary malignancies in patients with head and neck cancer.

## 4. Conclusions

The association between tobacco and alcohol consumption and the development of head and neck cancer and subsequent metachronous primary malignancies underscores the importance of patient education in smoking cessation and alcohol reduction. Implementing targeted preventive strategies may contribute to lowering the incidence of multiple primary cancers.

Routine follow-up and post-treatment surveillance play a crucial role in the early detection of second primary malignancies. In particular, bronchoscopy should be considered for patients presenting with suspected lung metastases during head and neck cancer follow-up to facilitate an accurate differential diagnosis.

Our case highlights the significance of personalized treatment approaches, including continuation and switch maintenance chemotherapy, in improving patient quality of life and extending survival. The long-term management of such cases benefits from a multidisciplinary approach, integrating oncology, thoracic surgery, and pathology expertise to optimize disease control and therapeutic outcomes.

## Figures and Tables

**Table 1:** Summary of the clinical evolution.

Date	Diagnosis	Key Findings	Treatment	Outcome
2017	Nasopharyngeal cancer	Stage III	Concurrent curative radiochemotherapy	Stable disease
2019	Chronic venous disease	Long-term anticoagulation	Anticoagulants	Managed
2020	Severe COVID-19	Respiratory complications	Supportive care	Recovered
2021	Lung cancer	Stage IV (M1PUL)	Chemotherapy, radiotherapy	Partial response, ongoing treatment
2022	Chronic osteomyelitis	*Staphylococcus aureus*	Antibiotics	Resolved

## Data Availability

Data are available upon request from the corresponding author.
